# Dual site external validation of artificial intelligence-enabled treatment monitoring for neovascular age-related macular degeneration in England

**DOI:** 10.1038/s41433-025-04025-4

**Published:** 2025-09-19

**Authors:** Henry David Jeffry Hogg, S. James Talks, Justin Engelmann, Marion Dawn Teare, Michael Pogose, Praveen J. Patel, K. Balaskas, G. Maniatopoulos, P. A. Keane

**Affiliations:** 1 University Hospitals Birmingham NHS Foundation Trust, Birmingham, UK; 2 Institute for Inflammation and Ageing, University of Birmingham, Birmingham, UK; 3 Moorfields Eye Hospital NHS Foundation Trust, London, UK; 4 Newcastle Eye Centre, Newcastle upon Tyne Hospitals NHS Foundation Trust, Newcastle upon Tyne, UK; 5 Institute of Ophthalmology, University College London, London, UK; 6 Population Health Sciences Institute, Newcastle University, Newcastle upon Tyne, UK; 7 Hardian Health, Haywards Heath, UK; 8 NIHR Biomedical Research Centre at Moorfields Eye Hospital and UCL Institute of Ophthalmology, London, UK; 9 School of Business, University of Leicester, Leicester, UK

**Keywords:** Retinal diseases, Medical imaging

## Abstract

**Background:**

Monitoring neovascular age-related macular degeneration (nAMD) is a significant contributor to ophthalmology demands in the NHS, with clinical capacity struggling to meet the demand. This task depends upon interpreting retinal optical coherence tomography (OCT) imaging, where artificial intelligence (AI) could rebalance clinical demand and capacity. However, evidence of safety and effectiveness in nAMD monitoring is lacking.

**Methods:**

Using a published non-inferiority design protocol, 521 pairs of ipsilateral retinal OCTs from consecutive visits for nAMD treatment were collected from two NHS ophthalmology services. Real-world binary assessments of nAMD disease activity or stability were compared to an independent ophthalmic reading centre reference standard. An AI system capable of retinal OCT segmentation analysed the OCTs, applying thresholds for intraretinal and subretinal fluid to generate binary assessments. The relative negative predictive value (rNPV) of AI versus real-world assessments was calculated.

**Results:**

Real-world assessments of nAMD activity showed a NPV of 81.6% (57.3–81.6%) and a positive predictive value (PPV) of 41.5% (17.8–62.3%). Optimised thresholds for intraretinal fluid increase (>1,000,000 µm³) and subretinal fluid increase (>2,000,000 µm³) for the AI system assessments produced an NPV of 95.3% (85.5–97.9%) and PPV of 57.8% (29.4–76.0%). The rNPV of 1.17 (1.11–1.23) met predefined criteria for clinical and statistical superiority and accompanied an rPPV of 1.39 (1.10–1.76).

**Conclusions:**

This study suggests that the same thresholds for interpreting OCT-based AI analysis could reduce undertreatment and overtreatment in nAMD monitoring at different centres. Interventional research is needed to test the potential of supportive or autonomous AI assessments of nAMD disease activity to improve the quality and efficiency of services.

## Introduction

Macular disease is the commonest primary diagnosis coded against NHS ophthalmology outpatient appointments, and ophthalmology is in turn the greatest contributor to NHS hospital outpatient demand [1]. Most macula service appointments concern the delivery of costly sight-preserving treatment (listed in the British National Formulary at around £800/unit), the most common indication being neovascular age-related macular degeneration (nAMD) [2]. Patients with nAMD receive treatments 4-12 times per year with treatment paradigms varying between NHS providers, but most commonly reflecting the Treat and Extend (TEX) protocol [3]. TEX and other treatment paradigms determine the treatment interval for individual patient’s largely based on biomarkers in macular optical coherence tomography (OCT). With current workforce capacity shortages in ophthalmology, there are already concerns over avoidable sight loss through delays in meeting these treatment needs [4, 5]. Variation in nAMD patient outcomes between services is reported but poorly characterized [6]. It is not clear if any differences in outcomes are driven by sociodemographic factors, though recent qualitative work suggests that greater distance from services, higher levels of comorbidity and lower levels of wealth and social support may negatively affect patients’ ability to travel to access care [7]. Besides any such inequities in current service provision, clinical risk presented by demand on services is set to grow with the increasing prevalence of age-related diseases and continuing workforce challenges in NHS ophthalmology. Recent NHS workforce data indicated that 75% of departments have insufficient consultant staffing for current needs and 28% of ophthalmologists (the highest among all specialties) leave NHS practice within 5 years of joining the specialty register [8, 9]. The economic impact of this capacity-demand mismatch will be non-linear as the annual non-drug cost per nAMD patient of service delivery has been estimated at £845, compared to £13,960 when a service’s capacity is exceeded [10].

Artificial intelligence (AI)-enabled analysis of retinal OCTs is a frequently cited hope to address the demand-capacity imbalance in nAMD care [11]. A collaboration between Moorfields Eye Hospital NHS Foundation Trust (MEH) and industry produced one of the earliest AI systems to meet this need [12]. Initial evidence focused on the potential to support triage of retinal referrals to ophthalmology departments, but treatment monitoring represents another high-volume target for AI innovation [1]. The technical validity of this technology and others for OCT analysis is well evidenced, but there is limited evidence at present for their clinical and cost effectiveness in real-world treatment monitoring applications [13–15]. The novelty of AI-enabled nAMD treatment monitoring may pose potential for new and much-needed efficiency gains for services, but it also presents clinical risk, which all stakeholders in nAMD require assurance on [7]. As early adopters within the NHS explore regulated AI products to deliver this functionality, the question of whether and how they can safely address the problem of demand-capacity imbalance in real-world care remains open [16].

This observational non-inferiority study represents the first published comparison of autonomous AI-enabled treatment monitoring decisions with standard care from two distinct NHS services. Using a diagnostic accuracy design, published with open access in a preceding protocol, it aims to test the potential of AI to safely improve the efficiency of NHS nAMD care [17]. The evidence is intended to inform early adopters of comparable regulated products and to guide the development of future products to maximise the value proposition for NHS services [16].

## Methods

This study is reported in line with the MI-CLAIM checklist (Supplementary Table 1) [18]. Details regarding the development of the AI system and validations in adjacent use cases are reported elsewhere [12, 13, 19]. This study adheres to the non-inferiority study design previously published, which used data from Newcastle Eye Centre (NEC) [17]. This report includes a replication study using the same protocol to include a second independent validation dataset drawn from MEH. Both datasets are of adequate size to independently satisfy the requirements of the power calculation set out in the protocol [17]. Curation of the second dataset from MEH was performed by the INSIGHT Health Data Research Hub and did not draw on imaging used to train the AI system [20]. Data from MEH include OCTs from distinct imaging equipment (Topcon™, multiple equipment models) than that used at NEC (Heidelberg Spectralis™). To summarise the design, sequential pairs of clinic visits for nAMD patients receiving treatment were randomly selected from the electronic medical record (EMR) of both institutions, applying eligibility criteria set out in the protocol. At NEC screening against eligibility criteria was performed manually by an ophthalmologist with clinical experience of the local EMR (Supplementary Table 2), whilst at MEH the eligibility criteria were applied as part of the search criteria by informaticians in the INSIGHT team (Supplementary Table 3) [20]. OCT data were sent in TIFF and DICOM formats respectively, from NEC and MEH for AI analysis, dictated by the export function of the OCT Picture Archiving and Communication System (PACS) at both institutions and the interoperability of the AI system. NEC imaging protocols produce 25 b-scan OCT volumes, but the AI system requires 49, so a preprocessing step to duplicate 24 of these b-scans was taken. No other pre-processing took place.

Binary assessments of disease activity (stable/improving versus worsening) from real-world care were inferred from changes in the planned treatment interval for each pair of clinic visits and free-text EMR entries where available (NEC only). A reference standard was produced by the blinded review of OCTs and basic clinical data by Moorfields Ophthalmic Reading Centre using a grading protocol reflective of standard NHS care (Supplementary Text 1). Imaging from each clinic visit was then processed by the AI system to produce volumes of various retinal tissue groups, including subretinal hyper-reflective material (SHRM), intraretinal fluid (IRF) and subretinal fluid (SRF). A range of thresholds based on clinical practice was applied for changes in IRF and SRF between the paired OCTs to derive binary assessments of disease activity. These thresholds were explored independently on the initially curated NEC dataset, before testing for generalisability on the MEH dataset. Estimates of diagnostic accuracy statistics were derived for these thresholds and for real-world care with 95% confidence intervals (CI) derived with the Clopper-Pearson method [21]. This CI method was chosen to enable a conservative interpretation of findings. The explorative approach to threshold design was due to the absence of evidence of how quantitative AI assessments of OCT biomarkers map to the established clinical paradigm of qualitative assessment from clinicians. The thresholds tested were designed to be memorable as heuristics. A single set of IRF, SRF and SHRM thresholds optimised to improve positive predictive value (PPV) without compromising the associated negative predictive value (NPV) was selected to take forward to derive the primary outcome measure. Negative predictive value (NPV) reflects the amount of sight-threatening ‘undertreatment’ that patients may experience from assessments of nAMD disease activity and aligns with stakeholders of nAMD care’s priority [7]. As per the protocol, the primary outcome was relative NPV (NPV of AI-led assessments divided by NPV of real-world assessments) with a non-inferiority requirement for the lower bound of the 95% confidence interval of the estimate to exceed 0.9 and a superiority requirement for the same lower bound to exceed 1.11 [17]. Metrics of technical performance, e.g., F1 score, were not relevant to the research question and therefore not included.

To explore if this stepwise heuristic approach to determining biomarker thresholds compromised the potential performance of AI-led assessments of disease activity, regression and random forest (RF) models were trained to predict disease activity from the IRF, SRF and SHRM volumes produced by the AI system. The 521 cases were randomly split into training (80%) and internal validation (20%) datasets, stratified by disease activity. Two machine learning approaches were implemented: Ridge-regularized Logistic Regression, a linear model, and Random Forest, a non-linear tree-based method. Input features were normalized to zero mean and unit variance.

Machine learning models output continuous risk scores rather than binary predictions. To compute NPV and PPV, these scores must be converted to binary predictions using a threshold. We evaluated three thresholding strategies: 0.5 probability (standard), matching the optimal heuristic rule’s NPV, and matching its PPV. This allowed direct comparison between machine learning predictions and our proposed heuristic rule.

Error analysis was performed with a quantitative approach of comparing the accuracy of AI-led assessments of disease activity between age, sex and ethnic subgroups. A qualitative approach was also taken for each case where AI-led assessments made an error that was not made in real-world care. Here, segmentation outputs were assessed by an ophthalmologist. The distribution of errors between different population subgroups was also subject to an exploratory analysis to check for foci of algorithmic bias.

## Results

Five-hundred and twenty-one eligible pairs of consecutive clinical visits were curated from 468 unique individuals at NEC (*n* = 262) and MEH (*n* = 259) (Table 1). The prevalence of disease activity at clinic visits determined by the reference standard was 22.8% (*n* = 59) and 27.1% (*n* = 71) at MEH and NEC, respectively (25.0% on aggregate). PPV for real-world assessments of disease activity was 40.4% (95% CI 16.8–61.8%) and 42.2% (30.0–54.2), with NPV of 82.2% (58.0–91.4%) and 80.8% (71.3–90.4) at MEH and NEC, respectively (Supplementary Table 4). Across both datasets, this produced aggregate estimates for PPV of 41.5% (17.8–62.3%) and NPV of 81.6% (57.3–81.6%).

**Table 1:** Characteristics of datasets from Moorfields Eye Hospital (MEH) and Newcastle Eye Centre (NEC).

		MEH (*n* = 259)	NEC (*n* = 262)
Sex	Female	166 (64.1%)	161 (61.5%)
	Male	93 (35.9%)	101 (38.5%)
Laterality	Left	120 (46.3%)	122 (46.6%)
	Right	139 (53.7%)	140 (53.4%)
Ethnicity recorded in EMR	White	171 (66.0%)	243 (92.7%)
	Asian	29 (11.2%)	1 (0.4%)
	Redacted	39 (15.1%)	0 (0.0%)
	Unknown	20 (7.7%)	18 (6.9%)
Diabetic status in EMR	Diabetes diagnosis recorded	10 (3.9%)	49 (18.7%)
IVI agent	Aflibercept	259 (0.0%)	238 (90.8%)
	Ranibizumab	0 (0.0%)	24 (9.2%)
VA in ETDRS letters, median (IQR)		65 (50–75)	67 (55–74)
Age, median (IQR)		83 (75–86)	81 (76–85)
Prior treatment interval in weeks, median (IQR)		8 (6–9)	8 (6–8)

*IVI* Intravitreal Injection, *VA* Visual Acuity, *ETDRS* Early Treatment of Diabetic Retinopathy Study, *IQR* Interquartile Range, *EMR* Electronic Medical Record.

**Fig. 1:**
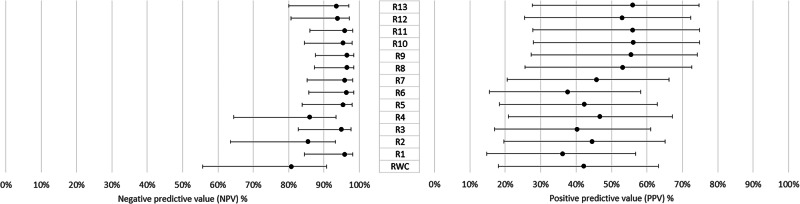
Positive predictive value and negative predictive value of real-world care (RWC) and 13 different heuristic rule sets applied to changes in AI system segmentation outputs for intraretinal fluid (IRF), subretinal fluid (SRF) and/or Subretinal hyper reflective material (SHRM) between clinic visit pairs. Error bars indicate 95% confidence intervals derived with the Clopper-Pearson method.

Sequential exploration of rule sets (Supplementary Text 2) was performed on the NEC dataset. This first established superior diagnostic accuracy performance for rule sets focusing on IRF and SRF without the use of SHRM as an input. Greater performance for rule sets using absolute rather than proportional thresholds for IRF and SRF change was then established. Finally, the absolute threshold for both IRF and SRF was increased in 10^6^ µm^3^ intervals until NPV was found to decline (Fig. 1). These rule sets were subsequently applied to the MEH dataset, where the patterns of performance were very similar in both datasets (Supplementary Table 5). On aggregate across all 521 cases, rule set 9 (R9) (>10^6^ µm^3^ of IRF increase or >2 × 106 µm^3^ SRF increase between visits) achieved an NPV of 95.3% (85.5–97.9%) and a PPV of 57.8% (29.4–76.0%) (Supplementary table 6). These performance statistics compared favourably to the Random Forest and Logistic Regression models, which were unable to exceed the PPV and NPV of R9 simultaneously at any threshold (Supplementary Table 7, Supplementary Fig. 1).

**Fig. 2:**
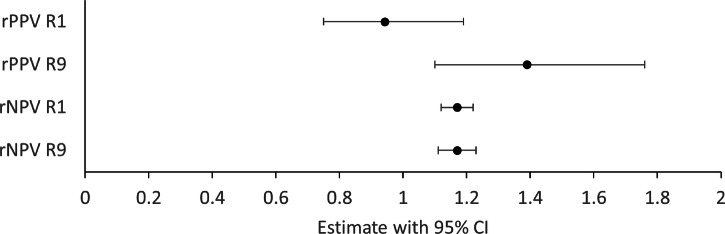
Forest plot of relative positive (rPPV) and negative (rNPV) predictive values for rule sets 1 (R1) and 9 (R9) applied to AI system segmentation outputs, relative to the diagnostic accuracy of real-world assessments of disease activity at Moorfields Eye Hospital and Newcastle Eye Centre (*n* = 521). The long-dashed line indicates the predetermined threshold for clinical non-inferiority (0.9), with the fine-dashed line indicating clinical superiority (1.11). Error bars indicate 95% confidence intervals (CI) derived with the method from Moskowitz et al. [17].

Comparing estimates of the diagnostic accuracy of AI-led and real-world clinical assessments of nAMD disease activity across both datasets using the simplest threshold for disease activity of any increase in IRF, SRF or SHRM for AI decisions (rule set 1, Supplementary Text 1) produced a rNPV of 1.17 (1.12–1.22) and a rPPV of 0.942 (0.75–1.19) (Fig. 2). Applying the thresholds described in R9 to determine AI-led assessments produced a comparable rNPV of 1.17 (1.11–1.23) and an improved rPPV of 1.39 (1.10–1.76). Estimates from all rule sets satisfy the primary endpoint to evidence non-inferior levels of sight-threatening undertreatment from AI-led decisions. When using R9, the rNPV estimate provides evidence of statistically and clinically significant improvement in this undertreatment. Estimates of rPPV also evidence the potential to reduce the amount of false positive assessments of nAMD and hence the levels of overtreatment through standard treatment protocols.

Across the 521 pairs of clinic visits, 15 false negative outputs from AI-led decisions using R9 occurred (5 NEC and 10 MEH). Four (26.7%) of these cases were correctly identified as demonstrating disease activity in real-world care (2 NEC and 2 MEH). Eighty-four false positive outputs from AI-led decisions using R9 occurred (53 NEC and 31 MEH). Fifty-three (63.1%) of these cases were correctly identified as demonstrating disease stability in real-world care (18 MEH and 35 NEC). The distribution of errors from R9 did not appear to differ significantly between subgroups defined by age, sex, ethnicity or diabetic status (Table 2).

**Table 2:** Accuracy of rule set 9 applied to AI system segmentation outputs for determining disease activity over subgroups defined by age, sex, ethnicity and diabetic status.

Characteristic		*n*	TP	TN	FP	FN	Accuracy	95% CI
Age	58–75	130	28	78	20	4	81.5%	74.9–88.2%
	75–82	130	30	73	23	4	79.2%	72.3–86.2%
	82–86	131	27	76	24	4	78.6%	71.6–85.6%
	86–98	130	30	80	17	3	84.6%	78.4–90.8%
Sex	Male	194	41	114	32	7	79.9%	74.3–85.5%
	Female	327	74	193	52	8	81.7%	77.5–85.8%
Diabetic	Yes	59	11	33	14	1	74.6%	63.5–85.7%
	No/not known	462	104	274	70	14	81.8%	78.3–85.3%
Ethnicity	White	414	96	235	72	11	80.0%	76.1–83.8%
	Asian	31	5	22	3	1	87.1%	75.4–98.9%
	Other	39	4	27	6	2	79.5%	66.8–92.2%
	Unknown	38	10	23	3	2	86.8%	76.1–97.6%

*TP* True positive, *TN* True negative, *FP* False positive, *FN* False negative, *CI* Confidence interval.

**Fig. 3:**
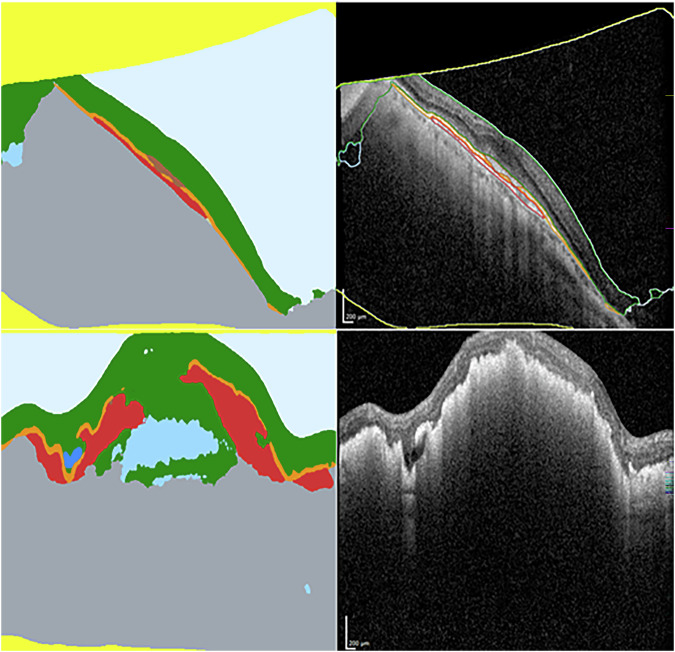
AI system segmentation outputs (left) paired with OCT b-scan inputs (right). Oblique orientation of retina and mirroring artefact (top) associated with non-anatomical attribution of intraretinal fluid (IRF). Large pigment epithelial detachment (PED) associated with non-anatomical attribution of IRF.

Imaging from the 57 cases where R9 led to errors not made in real-world care was reviewed. The cause of the error occurring between the MEH and NEC datasets appeared to differ. For the 37 errors that R9 led to in the NEC dataset, which were not made in real-world care, 18 (48.6%) involved segmentations of IRF or SRF that were immediately apparent as non-anatomical on clinical review (Fig. 3). All of these occurred in cases with suboptimal imaging quality on at least one b-scan. These imaging imperfections took the form of cropping artefacts, low illumination or a grainy appearance of the B-scan. On review of the 20 MEH error cases for R9, which were not made in real-world care, no such striking segmentation errors were found to have caused the errors, even though examples of poor imaging quality were present (e.g., no reflectance on several adjacent b-scans). On the MEH dataset, a subtle non-anatomical segmentation error of SRF applied to areas of atypical vitreoretinal interface was observed, e.g., from epiretinal membrane. The SRF volumes associated with this were not large enough to drive errors, which mainly came about from variable allocation of IRF to areas of diffusely thickened retina on poorer quality b-scans, resulting in apparent changes in IRF volume between scans, independent of actual changes. Considering populations who may be underserved by the AI system or similar technologies, the number of errors across the datasets permitted only an explorative analysis. No clear patterns were noted in the MEH dataset, but among the errors in the NEC dataset, there were three thin retinae, with a typical myopic appearance, and nine cases with large pigment epithelial detachment (Fig. 3).

## Discussion

### Principal findings

This study provides the first insights into the effectiveness and efficiency of AI-enabled treatment monitoring for nAMD relative to current NHS services. Using an internationally recognised ophthalmic reading centre as a reference standard, similar and substantial opportunities to improve on the amount of undertreatment and overtreatment from assessments of disease activity in nAMD care were identified at two independent NHS centres. This finding is at odds with the assumption that reading centres would ‘overcall’ disease relative to real-world care. However, it mirrors findings from a local independent consultant panel benchmarking process for a separate NHS nAMD service evaluation in Exeter [22]. The present study also found that the same thresholds of change in AI-derived volumes of IRF and SRF optimised diagnostic accuracy at both centres and that simple heuristic thresholds offer equivocal performance to thresholds derived through simple machine learning techniques. Whilst taking any increase of IRF, SRF or SHRM between clinic visits demonstrated non-inferior levels of sight-threatening undertreatment for AI compared to standard care, using higher thresholds for disease activity also enabled reductions in demand-generating overtreatment. AI-led assessments of nAMD disease activity and those made in real-world care commonly disagreed with the reference standard for different clinical cases. For decision support use cases, this presents an opportunity for further gains in diagnostic accuracy if modes of clinician-AI interaction can be designed that mitigate the risks in each individual approach, e.g., with the clinical team identifying unsuitable imaging quality and non-anatomical segmentations (Fig. 2) [23]. The AI system’s performance appears robust outside of its training dataset and subgroup analysis did not identify any concerns for biased performance between the different age, sex or ethnic groups presented.

### Comparison to wider literature

At the time of writing, three Artificial Intelligence as a Medical Device (AIaMD) products regulated for OCT analysis are listed on the UK Medicines and Healthcare products Regulatory Agency’s (MHRA) Public Access Registration Database (PARD) [24]. A few others are approved for similar use cases in other jurisdictions [16]. These all differ from the specific AI system evaluated in this study, but represent current opportunities to implement AIaMD for this use case in the NHS and healthcare services in other regulatory jurisdictions. A key similarity across these products is that they do not have regulatory approval for autonomous use cases, and so AI-enabled healthcare pathways for nAMD treatment monitoring must retain suitably qualified healthcare professionals as the responsible clinical decision maker [16]. This appears appropriate for the current evidence base, which focuses on the technical validity of segmentation outputs rather than the clinical validity of treatment decisions based upon those segmentations [25, 26]. However, evidence presented here suggests that the quantitative analysis of IRF and SRF enabled by AIaMD will not deliver its full potential without the application of non-zero decision thresholds, distinct from the existing paradigm of qualitative OCT interpretation in nAMD care. This need for non-zero decision thresholds to be used to deliver value from AIaMD in such use cases has been independently reported with other technologies [27]. If these thresholds are adequately simple, such as those presented here, then clinicians may choose to apply such thresholds to AIaMD outputs themselves to inform their practice as they do with other guidelines or heuristics, e.g., a threshold of 400 µm central subfield thickness to initiate treatment in diabetic macular oedema [28]. In doing so, they and their employing healthcare provider could expect to absorb more of the liability for any clinical errors that breach the duty of care they hold to their patients [29, 30]. This liability could be distributed more toward AIaMD manufacturers if thresholds (or the ability for users to set them) were to be incorporated into the AIaMD and their regulated intended use statements. Then, threshold-based nAMD treatment monitoring could more clearly fall within the intended use of the AIaMD, rather than the discretion of the clinical user. However, this would appear to represent a change in the intended use and risk profile against which currently available AIaMD achieved their regulatory approval, requiring new certification supported by an appropriate evidence base. In the current UK and European regulatory landscape, such a recertification process takes an approximate median of 18 months even when adequate clinical evidence is available [31]. This is due to the current capacity of regulatory authorities to follow their certification processes.

### Limitations

The assignment of ‘diagnostic error’ status to decisions made in real-world care is both reductionist and opaque within this retrospective study. It is entirely possible that so-called ‘undertreatment’ simply represented a holistic decision by a clinician delaying a follow-up appointment to accommodate competing demands on a patient’s time. This question over the study’s findings is partly mitigated by the apparent good performance of AI across all intuitive rule sets evaluated here, suggesting it is not simply over-fitting to the reference standard. It is, however, inherent to this retrospective study design, which was the most appropriate design given the lack of prior evidence to justify the clinical risk of an interventional study of AI-led assessment. Interventional research will be needed to determine the impact of any form of AI-enabled nAMD treatment monitoring on visual outcomes.

A key value proposition of AI-enabled care is presumed to be time-saving for users. Without simulated or actual AI-enabled clinical workflows, the present retrospective study had no means of measuring this. Given the relatively short period of time clinicians typically spend assessing OCT imaging once displayed, it seems unlikely that decision support AIaMD will reduce the clinician time required to review an individual case. If such efficiencies do arise, they will likely come from the platforms on which AIaMD is hosted rather than the AI technologies themselves. Autonomous use cases of future AIaMD may however, come to reduce the clinician time spent on individual reviews. The precedent for such autonomous products has recently been set with the certification of a class III AI-enabled dermatoscopic image interpretive software, listed on PARD [24]. This study illustrated a separate potential efficiency saving at the service level, rather than the individual case level. This efficiency saving is directly attributable to the AI technology and comes from the reduction of overtreatment in a nAMD service (measured by PPV). This value proposition will clearly depend on how users incorporate AIaMD outputs into their decision-making and may not be stable over the course of patients’ treatment. Clarity on this value proposition will require interventional evaluation.

The MEH dataset substantially improved on the ethnic diversity offered by the NEC dataset. However, the ethnic diversity of the validation data remains low overall. Similarly, the absence of labels for high myopia, a characteristic identified in some failure cases, limits the assurance this study can provide on robust performance, which must be addressed in future evaluations.

### Further work

A strength of this external validation study is its inclusion of two independent centres, using different OCT imaging equipment suppliers, compared to the same reference standard. To confirm the generalisability of the potential to improve PPV and NPV with this AI system and the decision thresholds specified, further replication studies at different sites would be valuable. Similarly, replication of the study with different AIaMD for OCT segmentation would also be valuable to understand if and how the value proposition of these technologies differs and whether or not optimal decision thresholds are consistent across them.

With current AIaMD approved only for decision support purposes, human-computer interaction and cognitive biases will hold a strong influence over the clinical impact of their use [32]. There is little evidence to understand this influence or what kinds of user training or workflows may help to optimise it [23]. Simulated or interventional studies of AI-enabled nAMD treatment monitoring will be required if patients and services are to unlock the value of AIaMD currently available for use [22].

To fully deliver the value proposition of AI-enabled nAMD treatment monitoring, it may be that AIaMD with regulatory approval for decision thresholds to be applied to segmentation outputs are required, potentially with approval for those outputs to be acted upon without immediate clinician review of each output. Whilst patient acceptance of autonomous AI decisions for their real-world care is largely untested, recent qualitative research suggests that patients and other stakeholders would find autonomous contributions from AI to nAMD treatment decisions acceptable if appropriately validated [7]. Interventional clinical trials and implementation research will be required to generate this evidence, to be followed by regulatory submissions that include an appropriate intended use statement and enable clinical application [15, 33].

### Concluding remarks

This study highlights a replicable opportunity to reduce the clinical demand associated with patient care, without compromising the accuracy of disease activity detection, in NHS nAMD services. If AI-enabled nAMD treatment monitoring is to improve the quality and efficiency of NHS services, it will depend upon the application of quantitative decision thresholds to OCT segmentation outputs that take opportunities to safely increase the frequency of treatment interval extension. Such an approach may be feasible with existing AIaMD, but further evidence is required and future AIaMD with explicit regulatory approval for autonomous use may be beneficial.

## Summary

### What was known before

- Treatment monitoring and delivery for neovascular age-related macular degeneration (nAMD) incurs a substantial demand on NHS ophthalmology services.
- Artificial intelligence (AI) systems can segment biomarkers of retinal disease in optical coherence tomography imaging with expert-level performance.

### What this study adds

- In the two representative NHS services tested there was a consistent tendency to create more treatment appointments than a reference standard deemed necessary.
- Autonomous assessments of nAMD disease activity by AI can offer superior diagnostic accuracy than that observed in real-world care.
- Optimising AI-led assessments requires non-zero quantitative thresholds for OCT biomarker change, which were consistent between the two NHS services tested.

## Supplementary information

Supplementary materials

## Data Availability

The Moorfields Eye Centre dataset is available through the INSIGHT Health Data Research Hub based at Moorfields. The hub is an NHS-led initiative comprising more than 35 million routinely collected ophthalmic images linked to clinical records. Data is made available to approved researchers working for patient benefit (an access fee applies to cover compute and curation costs and provide a return to the NHS.) For more information: https://www.insight.hdrhub.org/.
