# Supplementary material for: Dual site external validation of artificial intelligence-enabled treatment monitoring for neovascular age-related macular degeneration in England
Source: Eye (Lond). 2025 Sep 19;39(16):2973–9. doi: 10.1038/s41433-025-04025-4 (PMC12583501; doi:10.1038/s41433-025-04025-4)

# Supplementary file

**Supplementary table 1:** MI-CLAIM checklist

**Supplementary table 2:** Exclusion criteria for Newcastle Eye Centre (NEC) dataset curation and the frequency with which criteria led to ineligibilities. IVI = Intravitreal injection, nAMD = neovascular age-related macular degeneration, VEGF = Vascular endothelial growth factor, OCT = Optical coherence tomography, VA = Visual acuity, PRN = Pro-re nata

**Supplementary table 3:** Causes and frequencies for inability to complete processing of clinical cases from Moorfields Eye Hospital delivered through INSIGHT data request. MORC = Moorfields Ophthalmic Reading Centre

**Supplementary table 4:** 2x2 tables for diagnostic accuracy of assessments of neovascular age-related macular degeneration (nAMD) disease activity made in real-world care at Moorfields Eye Hospital (MEH) and Newcastle Eye Centre (NEC) compared to an independent reference standard generated by Moorfields Ophthalmic Reading Centre (MORC)

**Supplementary text 1:** Reading centre grading protocol (Images redacted)

**Supplementary text 2:** Definitions of rule sets trialled to define disease activity or stability/improvement from changes in AI system segmentation outputs from sequential pairs of retinal optical coherence tomography images. IRF = Intraretinal fluid, SRF = Subretinal fluid, SHRM = Subretinal hyper-reflective material

**Supplementary table 5:** Negative predictive value (NPV) and positive predictive value (PPV) of real-world care (RWC) at Moorfields Eye Hospital (MEH) and Newcastle Eye Centre (NEC) and different rule sets applied to AI system segmentation outputs. CI = Confidence Interval

**Supplementary table 6.** 2x2 tables for diagnostic accuracy of assessments of neovascular age-related macular degeneration (nAMD) disease activity made by applying rule set 9 (R9) to Moorfields Eye Hospital (MEH) and Newcastle Eye Centre (NEC) data compared to an independent reference standard generated by Moorfields Ophthalmic Reading Centre (MORC).

**Supplementary table 7.** Negative predictive value (NPV) and positive predictive value (PPV) of rule set 9, real-world care (RWC), logistic regression and random forest models with different thresholding approaches on randomly selected test set (n=105).

**Supplementary figure 1.** Receiver Operator Characteristic (ROC) curve displaying diagnostic performance of rule set 9 (proposed rule), consultant-led care, logistic regression and random forest models. AUC = Area Under the Curve

## Supplementary table 1: MI-CLAIM checklist

| Section | Requirement | Completed: section | Notes |
| --- | --- | --- | --- |
| Part 1: Study design | The clinical problem in which the model will be employed is clearly detailed in the paper | Introduction |  |
|  | The research question is clearly stated. | Introduction |  |
|  | The characteristics of the cohorts (training and test sets) are detailed in the text | Methods | Development paper cited (De Fauw), test set characterised (table 1) |
|  | The cohorts (training and test sets) are shown to be representative of real-world clinical settings | Methods | Random sampling described and protocol (Hogg) cited |
|  | The state-of-the-art solution used as a baseline for comparison has been identified and detailed | Methods | Real-world clinical care |
| Parts 2 and 3: Data and optimization | The origin of the data is described and the original format is detailed in the paper | Methods | Further detail signposted in cited protocol |
|  | Transformations of the data before it is applied to the proposed model are described. | Methods |  |
|  | The independence between training and test sets has been proven in the paper. | Methods |  |
|  | Details on the models that were evaluated and the code developed to select the best model are provided. | Methods | Citation to development paper |
|  | Is the input data type structured or unstructured? | Methods | Imaging |
| Part 4: Model performance | The primary metric selected to evaluate algorithm performance (e.g., AUC, F-score, etc.), including the justification for selection, has been clearly stated. | Methods | Algorithm performance metric not used |
|  | The primary metric selected to evaluate the clinical utility of the model (e.g., V, NNT, etc.), including the justification for selection, has been clearly stated. | Methods | rNPV |
|  | The performance comparison between baseline and proposed model is presented with the appropriate statistical significance | Methods | Detailed in cited protocol but summarised here |
| Part 5: Model examination | Examination technique 1a | Results | Diagnostic accuracy stats |
|  | Examination technique 2a | Results | Qualitative error analysis + quantitative subgroup error analysis (table 2) |
|  | A discussion of the relevance of the examination results with respect to model/algorithm performance is presented. | Principal findings |  |
|  | A discussion of the feasibility and significance of model interpretability at the case level if examination methods are uninterpretable is presented. | Results |  |
|  | A discussion of the reliability and robustness of the model as the underlying data distribution shifts is included. | Principal findings and limitations |  |
| Part 6: Reproducibility | Tier 4 – no sharing |  | Model validation study, not development |

## Supplementary table 2: Exclusion criteria for Newcastle Eye Centre (NEC) dataset curation and the frequency with which criteria led to ineligibilities. IVI = Intravitreal injection, nAMD = neovascular age-related macular degeneration, VEGF = Vascular endothelial growth factor, OCT = Optical coherence tomography, VA = Visual acuity, PRN = Pro-re nata

For manual curation of the NEC dataset (described in prior protocol), 421 patients were screened on the to identify the 262 eligible clinic visits required by the power calculation

| Step number | Exclusion criteria | Ineligibilities for initially identified clinic visit n=159 (%) | No eligible case available for eye n=82 (%) |
| --- | --- | --- | --- |
| 1 | The visit is less than 10 weeks after the eye’s first IVI | 1 (0.6) | 0 (0.0) |
| 2 | The eye has a retinal diagnosis beside nAMD or is enrolled in a study | 44 (27.7) | 42 (51.2) |
| 3 | The visit does not involve anti-VEGF treatment for nAMD | 1 (0.6) | 1 (1.2) |
| 4 | The VA measurements of interest are likely influenced by other interventions | 3 (1.9) | 1 (1.2) |
| 5 | There is no consultation at this visit | 52 (32.7) | 15 (18.3) |
| 6 | The clinician does not state the treatment interval they intend | 1 (0.6) | 0 (0.0) |
| 7 | VA measurements for the visit and the prior one are unavailable | 0 (0.0) | 0 (0.0) |
| 8 | Co-located 25 slice fovea-centred OCTs are not available for the visit and the prior one | 29 (18.2) | 9 (11.0) |
| 9 | The visit is under the PRN protocol | 28 (17.6) | 14 (17.1) |

## Supplementary table 3: Causes and frequencies for inability to complete processing of clinical cases from Moorfields Eye Hospital delivered through INSIGHT data request. MORC = Moorfields Ophthalmic Reading Centre, OCT = Optical Coherence Tomography

As the MEH dataset was accessed via a data service request with eligibility criteria coded into a database query, 270 clinic visits were requested allowing for some attrition. Following receipt and processing of this dataset, 11 MEH clinic visits could not be fully processed, leaving 259 for analysis.

| Reason | Frequency (%) |
| --- | --- |
| MORC report an OCT from visit pair ungradable | 3 (27.3) |
| One AI system output from visit pair unavailable in INSIGHT export | 6 (54.5) |
| One OCT from visit pair unavailable in INSIGHT export | 2 (18.2) |

## Supplementary table 4: 2x2 tables for diagnostic accuracy of assessments of neovascular age-related macular degeneration (nAMD) disease activity made in real-world care at Moorfields Eye Hospital (MEH) and Newcastle Eye Centre (NEC) compared to an independent reference standard generated by Moorfields Ophthalmic Reading Centre (MORC)

|  |  | MEH – real world care | | NEC – real world care | |
| --- | --- | --- | --- | --- | --- |
|  | nAMD disease assessment | Active | Stable/ improving | Active | Stable/ improving |
| MORC | Active | 23 | 36 | 38 | 33 |
|  | Stable/ improving | 34 | 166 | 52 | 139 |

## Supplementary text 1: Reading centre grading protocol (Images redacted)

**General Notes for OCT Image Grading**

Spectral domain OCT will be utilised to assess subretinal fluid, intraretinal fluid, abnormalities within the retinal layers and neovascular lesion components at each visit.

Maximum and foveal measurements can be taken on any of the line scans present. Volume measurements and segmentation information must be taken from volume or cube scans.

**Overview of grading**

It has been agreed that all imaging modalities should be used to determine a final grading. This is important to produce coherent grading data for the imaging modalities being graded or assessed in the case of the other types of images e.g. colour images.

You are permitted to go back to check any type of images available and change grading data if you realise that you have graded incorrectly.

**It is very important to remember however, that you can only grade what you see.** For example, if a certain lesion characteristic is identified in the additional image but cannot be identified on any of the OCT images then that feature should not be graded as present on the OCT grading form. If, however, a grader suspects SRF on additional modality but confidence level is less than 90%, and the OCT confirms the presence of SRF, the grader can modify the selection.

**Scan Quality Assessments**

Is the OCT scan profile complete?

The OCT scan profile for TEMS participants varies depending on the OCT system used. The following table details scans required per acquisition system:

Scans Required:

Heidelberg Spectralis SD OCT- Volume Scan, variable b-scan density (range 7-49)

Grading task: Comparison of sequential volume SD-OCT scans (two scans/patient) under management with anti-VEGF treatment for nAMD.

Classification options from the sequential scan comparison with respect to disease activity:

**‘same’ ‘worse’ and ‘better’**

**Overall scan quality**

Scan Quality assessment is not a formalised datafield for the TEMS study. It was nevertheless reviewed by expert graders and notes were added for cases with poor gradeability.

An overall assessment of the scans provided should be made and described as below:

**Good**

Excellent or good discrimination of retinal layers and sub retinal layers (particularly External Limiting Membrane (ELM), Ellipsoid Zone(EZ) and RPE/Bruch’s Complex(R/BC)) throughout the majority of the scans supplied especially in sub foveal region.

**Fair**

Discrimination of retinal layers and sub-retinal layers (particularly External Limiting Membrane, Ellipsoid Zone and RPE/Bruch’s Complex) may be less distinct than ‘Good – above’ but still of suitable quality for acquisition of reliable data. This should be the case throughout the majority of the scans supplied especially in sub-fovea region.

**Poor**

Discrimination of retinal layers and sub retinal layers (particularly External Limiting Membrane, Ellipsoid Zone and RPE/Bruch’s Complex) is not distinct. Some information may still be discerned but no reliable measurements could be taken. This should be the case throughout the majority of the scans supplied especially in sub-fovea region.

**Ungradable**

Discrimination of retinal layers and sub retinal layers (particularly External Limiting Membrane, Ellipsoid Zone and RPE/Bruch’s Complex) is not of suitable quality for acquisition of reliable data. This should be the case throughout the majority of the scans supplied especially in sub foveal region.

**Identification of lesion components**

There are 135 pairs of OCTs from patients with nAMD. They are from two different visits of the same patient at the injection clinic.

Sequence of OCT scan review: OCT scans visualised on the Moorfields Grading Portal are reviewed, starting with the first/baseline OCT and followed by review of the second sequential OCT scan to make the determination if the disease activity is **better**, **stable** or **worse**.

**Some General AMD lesions assessment**

This a general information regarding some lesions that can be seen during a neovascular Age-related Macular Degeneration assessment.

**RPE/Bruch’s Complex, (R/BC)**

Graders should assess the **RPE/Bruch’s Complex**, (**R/BC**) and evaluate it for any fluctuations in thickness and/or continuity. If the grader deems the R/BC to be abnormal then the details will be captured. An increase in thickness of the R/BC may be seen in patients with AMD and is typically due to CNV, with associated fibrosis. The R/BC is thought to represent the RPE and choriocapillaris layers of the retina. Theoretically it should be possible to follow the R/BC along the scan starting from an area of normal thickness R/BC. By doing so it should be possible to tell whether the R/BC is thickened. If the R/BC appears increased in thickness on any scan, grade the presence of increased thickness as compared with the baseline visit scan as an auxiliary factor in ambiguous cases to determine if the disease activity is **better, stable, or worse**.

N.B. The thickening of the R/BC may be above or below the RPE. However, it is extremely difficult to be certain where the RPE actually is, particularly when the architecture is totally altered and distorted by advanced CNV. Thus, it is extremely difficult to usefully and reliably distinguish between classic/occult FPED/mixed CNV (e. g below). For the TEMS study the grader will not have to determine the nature of the R/BC thickening, just the presence or absence of thickening.

Redacted imaging

Decreased thickness of the R/BC may be due to atrophy of the RPE cells and adjacent choriocapillaris.

Due to increased penetration through atrophic neurosensory retina and RPE, the choroid may exhibit an area of increased optical reflectivity (e.g. below).

Redacted imaging

OCT shows a well-defined area of increased choroidal reflection due to decreased thickness of the R/BC

**RPE tear**

An RPE tear appears as an area of high reflection from the choroid due to the increased penetration of the probe light into the choroid as a result of absence of overlying RPE with an adjacent, thick, elevated area of high reflectivity which corresponds to the folded-up layer of RPE. The choroidal reflection below the area of rolled-up RPE is completely shadowed because of decreased penetration of the probe light through multiple layers of RPE rolled up.

RPE tears typically develop along the edge of a PED, where there is increased tension along the RPE caused by the elevation. Tractional pull along the RPE, particularly by fibrovascular tissue can create enough force for the RPE to rip along the edge. Often this process is quite traumatic and causes a lot of subretinal and sub RPE haemorrhage in the acute stage with associated, and often substantial, loss of vision, particularly if these changes affect the centre of the fovea. If an RPE tear has occurred in one eye, there is an increased risk that it could also occur in the fellow eye at some time in the future.

Redacted imaging

*OCT shows discontinuity of the R/BC due to an RPE tear (white arrow). The area torn and rolled back is seen elevated whilst the area devoid of RPE has increased choroidal reflectance. Fundus picture shows the orientation of the scan through the RPE tear. Red arrow shows the extent of the atrophy.*

Development of an RPE tear represents disease worsening for TEMS grading.

Elevation of the R/BC represents a pigment epithelial detachment (PED). There are a number of different types of PED which can be identified on OCT.

**Serous Pigment Epithelial Detachment (SPED)**

On OCT a SPED appears as focal elevation of the R/BC over an optically clear space with sharp margins. The detached RPE is slightly more reflective than normal (possibly due to morphological changes in detached RPE). Increased reflectivity of the R/BC reduces reflection from the underlying choroid. The amount of shadow will depend on the amount of sub-RPE fluid present and the height of the SPED. The angle at the edge of the detachment is typically acute, probably because of the tight adherence of RPE cells to Bruch’s membrane at the edge of the detachment.

Redacted imaging

OCT shows elevation of the outer margin of the R/BC with a non-reflective space beneath (consistent with the appearance of a SPED) and an area of non-reflectivity between the retina and R/BC (consistent with the presence of SRF)

SPED presence and/or change is not a feature considered for the determination of disease activity for the TEMS study.

**Haemorrhagic / Pigmented PED**

These two types of PED can be distinguished from other PEDs by the presence of a thin, moderately reflective layer directly beneath the detached/elevated R/BC. Blood directly underneath the RPE is only moderately reflective because of attenuation of probe light through detached RPE. Likewise pigment only allows a certain amount of penetration of the OCT probe light.

Penetration through blood is usually less than 100μm. Reflections from deeper portions of blood and choroid are severely attenuated when either blood or heavily pigmented scarring is present.

Loss of choroidal details correlates with thickness of the blood/pigment. It is extremely difficult to differentiate between blood and pigment on OCT, so a heavily pigmented and elevated scar will have similar OCT appearances to a densely haemorrhagic PED,

Redacted imaging

OCT and corresponding colour image showing haemorrhagic PED.

Development of a haemorrhagic PED represents disease worsening for TEMS study grading.

**Fibro-vascular Pigment Epithelial Detachment (FPED)**

There is well-defined irregular and relatively shallow elevation of the R/BC with a deeper area of mild backscattering corresponding to fibrous proliferation.

There is moderate reflectivity throughout entire sub-RPE (sub-R/BC) space. Fibrovascular proliferation does not scatter as much light compared to blood and therefore allows for penetration of light through entire lesion down to the level of choroid, where attenuation of choroidal reflection is typically noted.

Redacted imaging

OCT shows elevation of the outer margin of the R/BC (FPED) with an area of increased backscattering corresponding to fibro-vascular material.

FPED alone (in the absence of change in SRF/IRF) is not taken into consideration to determine change in disease activity at the TEMS study.

**Drusenoid PED**

Drusenoid PED occurs when a number of soft drusen become confluent with each other. It presents on OCT as focal elevation of the R/BC, which may be typically found under the fovea, with usually a very uniform area of moderately reflective material underneath.

Redacted imaging

OCT shows elevation of the outer margin of the R/BC with an area of uniform moderate reflectivity below (drusenoid PED). This feature is not considered to determine disease activity at the TEMS study.

**SRF – Sub retinal fluid**

Areas of hypo-reflectivity separating the neurosensory retina from the R/BC (representing SRF)

**Non-reflective Fluid**

An optically clear space anterior to the R/BC. The space represents a collection of fluid that can be evaluated quantitatively.

Subretinal fluid (SRF) is identified when a non-reflective space between the posterior boundary of neurosensory retina and the R/BC is present. The R/BC is undisrupted and follows the contour of globe (in distinction to intraretinal oedema the non-reflective area of SRF is not present within neurosensory retina, nor is it typically round). SRF non-reflective space is typically semi-circular with tapered lateral extensions. Numerous variations may present due to the presence of other abnormalities such as CNV or PEDs.

Redacted imaging

OCT shows an area of no reflectivity between the neurosensory retina and R/BC

**Moderately reflective Fluid**

Areas of moderate hypo-reflectivity could be observed in SRF. These may be due to blood or turbid fluid. They will not usually be as hypo reflective as sub-retinal fluid (some evidence of reflectance will be present) but will be present in the space between the neurosensory retina and R/BC.

Redacted imaging

OCT shows an area of moderate reflectivity between the neurosensory retina and R/BC

**Key feature for classifying change in disease activity for the TEMS study.**

**Hypo-reflective spaces within the neurosensory retina – Intraretinal Fluid (IRF)**

Macular oedema presents on OCT as optically clear spaces in the intra-retinal layers and can be focal or diffuse. It can be easily missed on interpretation of FA alone (due to overwhelming fluorescence arising from CNV).

On OCT, cystoid macular oedema (CMO) is visualised as intra-retinal, hypo-reflective spaces with no reflectivity (appear black) due to the presence of intraretinal fluid. Hypo-reflective spaces are typically separated from each other by reflective septa.

Hypo-reflective spaces are typically round/oval in shape and must have a minimum height of at least 50μm. Small hypo-reflective spaces are generally confined to the outer retinal layers while larger hypo-reflective spaces can span nearly the entire thickness of the retina. CMO may present as one hypo-reflective space only or as multiple hypo-reflective spaces.

Breakdown of the reflective septa causes disruption of the normal retinal architecture and enlargement and coalescence of the hypo-reflective spaces. This is more likely to occur if CMO becomes chronic and longstanding and this disruption to the architecture then becomes permanent. Diffuse oedema cannot be easily seen or measured.

Redacted imaging

OCT shows focal areas hypo-reflective spaces within the neurosensory retina

Redacted imaging

OCT shows a very oedematous retina with diffuse hypo-reflective spaces.

**Key feature for classifying change in disease activity for the TEMS study.**

**Hyper-Reflective Material**

One recently identified OCT feature in nv-AMD is hyper-reflective material that obscures normal retinal anatomical features including the disruption of layers. These are referred to as hyper-reflective material (HRM). HRM can have varying hyper-reflectiveness within its boundaries and will be distinguishable from surrounding neural components. It can be well-defined or ill-defined. HRM in isolation is not considered to determine disease activity for TEMS.

Redacted imaging

Sub-retinal HRM

Redacted imaging

Sub-retinal HRM

**Other pathology present**

Graders should assess all scan available for other pathologies and record presence. If a pathology not listed is present, the grader should select ‘other’ and then add a comment. These pathologies and their OCT manifestations should not be confused as determinants of nAMD disease activity at the TEMS study.

**Macular Hole**

A macular hole typically appears as a full-thickness central defect with neurosensory retinal thickening at the hole margins. Often there are hypo reflective spaces observed at the hole margins within the thickened retina and the inner retina at the hole margin appears slightly elevated. An epiretinal membrane (which is a highly reflective layer overlying the inner retina) may be seen surrounding the macular hole.

Redacted imaging

Macular Hole

**Epiretinal membrane (ERM)**

ERM typically appears as a highly reflective layer visible on the inner retinal surface. Essentially an ERM is demonstrated by a thickening of the normally thin highly reflective layer on the surface of the retina. This represents the nerve fibre layer in the normal eye, and it is thicker on the side next to the disc than it is temporal to the fovea. In ERM this highly reflective band is abnormally thickened. There are different types of ERM, depending on the degree of attachment or separation of the abnormally thickened band, which basically represents fibrous tissue running along the surface of the retina, which can then become detached from the surface of the retina in places, causing a separation. Also the fibrotic band can contract causing surface wrinkling of the inner highly reflective band.

Redacted imaging

Epiretinal membrane

**Evidence of Vitreo-Retinal Attachment**

This is defined as an incomplete separation of posterior hyaloid with focal attachment at the macula/fovea. This is only demonstrable if the correct slice through the precise point of attachment is taken, and any OCT slice or section taken which is outside of this point will miss it totally. The posterior hyaloid face, if apparent at all, is only visible if there has been some separation or detachment of the vitreous from the retinal surface. It appears as a thin highly reflective line within the vitreous and is usually just above and parallel to the inner surface of the retina on the OCT scan. In some disease states, for example diabetes, this posterior hyaloid face becomes thickened and more prominent on the OCT. Grade as present only if you actually see point of attachment between posterior hyaloid face and macula on any OCT scan. The options for grading are:

Redacted imaging

Vitreous attached at macula with traction

Redacted imaging

Vitreous attached at macula without traction

Redacted imaging

Vitreous definitely detached at the fovea

**Retinal Layers on OCT**

Redacted imaging

The above diagram must be used by all grading staff for the identification of the retinal layers described in the definitions.

**Grading for TEMS**

Pairs of OCTs from patients with **neovascular AMD**. They are from two different visits of the same patient at the injection clinic.

The grader should review the first OCT and then review the recent 'study' OCT to decide if the disease activity is **better, stable or worse.**

**Examples of grading decisions post-adjudication by K Balaskas.**

**Change (better): Resolution of SRF and IRF**

Redacted imaging

Redacted imaging

**Change (better): Significant reduction in SRF and SHRM**

**No change (stable): Minor reduction in SRF < 10%**

Redacted imaging

**No change (stable): FPED without SRF/IRF in either visit**

Redacted imaging

Redacted imaging

**No change (stable): minor degenerative cystic changes overlying atrophy <10 microns in diameter at baseline, not observed in second visit. Not disease activity-related change.**

**Change (better): Significant reduction in IRF**

Redacted imaging

Redacted imaging

**Change (worse): Expansion of cystic changes (IRF) towards the subfoveal area**

Redacted imaging

**No change (stable): Unchanged appearance of inner retinal hypo-reflective space (presumed IRF, although in this case it is more suggestive of degenerative cystic changes overlying an area of macular fibrosis rather than exudative IRF)**

## Supplementary text 2: Definitions of rule sets trialled to define disease activity or stability/improvement from changes in AI system segmentation outputs from sequential pairs of retinal optical coherence tomography images. IRF = Intraretinal fluid, SRF = Subretinal fluid, SHRM = Subretinal hyper-reflective material

1. Any increase in IRF, SRF or SHRM
2. Any increase in IRF
3. Any increase in IRF or SRF
4. > 10% increase in IRF
5. > 10% increase in IRF or SRF
6. > 10% increase in IRF, SRF or SHRM
7. > 10% increase in IRF or >1000000 µm^3^ increase in SRF
8. > 1000000 µm^3^ increase in IRF or >1000000 µm^3^ increase in SRF
9. **> 1000000 µm^3^ increase in IRF or >2000000 µm^3^ increase in SRF**
10. > 2000000 µm^3^ increase in IRF or >2000000 µm^3^ increase in SRF
11. > 1000000 µm^3^ increase in IRF or >3000000 µm^3^ increase in SRF
12. > 3000000 µm^3^ increase in IRF or >1000000 µm^3^ increase in SRF
13. > 3000000 µm^3^ increase in IRF or >3000000 µm^3^ increase in SRF

## Supplementary table 5: Negative predictive value (NPV) and positive predictive value (PPV) of real-world care (RWC) at Moorfields Eye Hospital (MEH) and Newcastle Eye Centre (NEC) and different rule sets applied to AI system segmentation outputs. CI = Confidence Interval

| MEH results | NPV | lower CI | upper CI | PPV | upper CI | lower CI |
| --- | --- | --- | --- | --- | --- | --- |
| RWC | 82.2% | 58.0% | 91.4% | 40.4% | 16.8% | 61.8% |
| R1 | 95.0% | 83.7% | 97.8% | 43.7% | 64.4% | 19.1% |
| R2 | 85.6% | 63.8% | 93.2% | 44.4% | 19.4% | 65.4% |
| R3 | 95.0% | 83.7% | 97.8% | 43.7% | 19.1% | 64.4% |
| R4 | 85.7% | 64.1% | 93.3% | 45.7% | 66.5% | 20.2% |
| R5 | 94.4% | 82.1% | 97.5% | 43.6% | 64.3% | 19.0% |
| R6 | 94.4% | 82.1% | 97.5% | 43.6% | 64.3% | 19.0% |
| R7 | 95.5% | 85.1% | 98.0% | 50.0% | 23.2% | 70.0% |
| R8 | 94.9% | 83.7% | 97.7% | 60.2% | 31.2% | 78.0% |
| **R9** | **94.4%** | **82.6%** | **97.5%** | **61.3%** | **32.1%** | **78.7%** |
| R10 | 94.5% | 82.8% | 97.5% | 63.6% | 34.2% | 80.3% |
| R11 | 94.5% | 82.7% | 97.5% | 62.8% | 33.5% | 79.8% |
| R12 | 94.5% | 82.8% | 97.5% | 63.7% | 34.2% | 80.3% |
| R13 | 94.1% | 81.9% | 97.4% | 66.7% | 37.2% | 82.4% |

| NEC results | NPV | lower CI | upper CI | PPV | lower CI | upper CI |
| --- | --- | --- | --- | --- | --- | --- |
| RWC | 80.8% | 55.7% | 90.7% | 42.2% | 18.1% | 63.2% |
| R1 | 95.9% | 84.5% | 98.2% | 36.2% | 14.8% | 56.8% |
| R2 | 85.5% | 63.5% | 93.2% | 44.5% | 19.6% | 65.2% |
| R3 | 94.9% | 82.7% | 97.7% | 40.2% | 17.0% | 61.0% |
| R4 | 86.0% | 64.4% | 93.4% | 46.7% | 20.9% | 67.1% |
| R5 | 95.3% | 83.8% | 97.9% | 42.3% | 18.3% | 63.0% |
| R6 | 96.3% | 85.7% | 98.4% | 37.6% | 15.5% | 58.3% |
| R7 | 95.8% | 85.3% | 98.1% | 45.8% | 20.5% | 66.3% |
| R8 | 96.4% | 87.2% | 98.4% | 53.2% | 25.6% | 72.6% |
| **R9** | **96.5%** | **87.6%** | **98.5%** | **55.5%** | **27.3%** | **74.3%** |
| R10 | 95.3% | 84.4% | 97.9% | 56.1% | 27.9% | 74.8% |
| R11 | 95.9% | 86.0% | 98.2% | 56.0% | 27.8% | 74.8% |
| R12 | 93.8% | 80.7% | 97.2% | 53.0% | 25.4% | 72.4% |
| R13 | 93.5% | 80.0% | 97.1% | 56.0% | 27.7% | 74.7% |

## Supplementary table 6. 2x2 tables for diagnostic accuracy of assessments of neovascular age-related macular degeneration (nAMD) disease activity made by applying rule set 9 (R9) to Moorfields Eye Hospital (MEH) and Newcastle Eye Centre (NEC) data compared to an independent reference standard generated by Moorfields Ophthalmic Reading Centre (MORC).

|  |  | MEH -R9 | | NEC – R9 | |
| --- | --- | --- | --- | --- | --- |
|  | nAMD disease assessment | Active | Stable/ improving | Active | Stable/ improving |
| MORC | Active | 49 | 10 | 66 | 5 |
|  | Stable/ improving | 31 | 169 | 53 | 138 |

## Supplementary table 7. Negative predictive value (NPV) and positive predictive value (PPV) of rule set 9 (R9), real-world care (RWC), logistic regression and random forest models with different thresholding approaches on randomly selected test set (n=105).

|  | PPV (%) | NPV (%) |
| --- | --- | --- |
| Logistic Regression (0.5) | 75.0 | 77.2 |
| Logistic Regression (PPV-matched) | 66.7 | 76.5 |
| Logistic Regression (NPV-matched) | 30.0 | 92.0 |
| Random Forest (0.5) | 66.7 | 82.2 |
| Random Forest (PPV-matched) | 61.9 | 84.5 |
| Random Forest (NPV-matched) | 44.7 | 91.4 |
| R9 | 62.5 | 91.8 |
| RWC | 35.7 | 79.2 |

## Supplementary figure 1. Receiver Operator Characteristic (ROC) curve displaying diagnostic performance of rule set 9 (proposed rule), consultant-led care, logistic regression and random forest models. AUC = Area Under the Curve


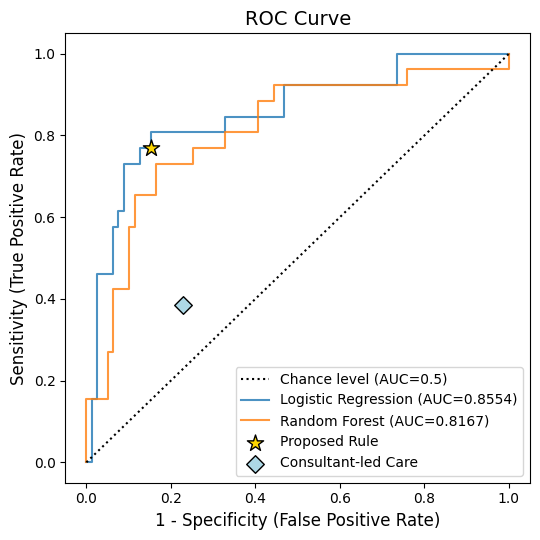

Supplement: Supplementary file 1 — Supplementary materials [file 41433_2025_4025_MOESM1_ESM.docx]
